# N-Acetylcysteine Inhibits Coxsackievirus B3 Replication by Downregulating Eukaryotic Translation Elongation Factor 1 Alpha 1

**DOI:** 10.3390/v16091503

**Published:** 2024-09-23

**Authors:** Yao Wang, Tian Luan, Lixin Wang, Danxiang Feng, Yanyan Dong, Siwei Li, Hong Yang, Yang Chen, Yanru Fei, Lexun Lin, Jiahui Pan, Zhaohua Zhong, Wenran Zhao

**Affiliations:** 1 Department of Cell Biology, Harbin Medical University, 157 Baojian Road, Harbin 150081, China; 2 Department of Microbiology, Harbin Medical University, 157 Baojian Road, Harbin 150081, China

**Keywords:** coxsackievirus B, N-acetylcysteine, eukaryotic elongation factor 1 alpha 1, autophagy

## Abstract

Group B Coxsackieviruses (CVB) are one of the causative pathogens of myocarditis, which may progress to cardiomyopathy. The pathogenesis of CVB is not fully understood, and effective antiviral therapy is not available. N-acetylcysteine (NAC), the classic antioxidant, has been used in clinical practice for several decades to treat various medical conditions. In this study, the anti-CVB effect of NAC was investigated. We show that NAC dramatically suppressed viral replication and alleviated cardiac injury induced by CVB3. To further study the antiviral mechanism of NAC, RNA-sequencing was performed for CVB3-infected cells with NAC treatment. We found that eukaryotic elongation factor 1 alpha 1 (*EEF1A1*) is one of the most upregulated genes in CVB3-infected cells. However, *EEF1A2*, the highly homologous isoform of *EEF1A1*, remains unchanged. *EEF1A1* expression was significantly suppressed by NAC treatment in CVB3-infected cells, while *EEF1A2* was not affected. eEF1A1 knockdown significantly inhibited CVB3 replication, implicating that eEF1A1 facilitates viral replication. Importantly, we show that eEF1A1, which was not expressed in the myocardia of newborn mice, was significantly upregulated by CVB3 infection. NAC markedly downregulated the expression of eEF1A1 but not eEF1A2 in the myocardia of CVB3-infected mice. Furthermore, NAC accelerated eEF1A1 degradation by promoting autophagy in CVB3-infected cells. We show that p62, one of the critical adaptors of autophagic targets, interacts with eEF1A1 and was downregulated in CVB3-infected cells upon NAC treatment. Taken together, this study demonstrated that NAC shows a potent anti-CVB effect through the downregulation of eEF1A1.

## 1. Introduction

Group B Coxsackieviruses (CVB) are known as the most common cause of viral myocarditis, which primarily affects children and young adults [1]. In some cases, myocarditis may lead to heart dysfunction with severe myocardial damage [2]. Viral myocarditis is also considered one of the common causes of dilated cardiomyopathy (DCM) [3]. Laboratory and clinical studies showed that viral clearance with interferon (IFN) treatment was associated with improved cardiac function [4]. Moreover, it has been implicated that eliminating cardiac virus load is crucial for blocking the progression of myocarditis to DCM [2]. However, currently, there are no approved antiviral therapies for viral myocarditis.

CVBs are non-enveloped viruses with single-strand positive-sensed genomic RNA [5]. Viral particles are shaped in an icosahedron structure with a diameter of approximately 30 nm [6,7,8]. CVB genomic RNA is about 7.4 kb with a single open reading frame (ORF) flanked by 5′- and 3′-untranslated regions (UTR) [9]. CVB encodes four structural proteins (VP1-VP4) and seven viral nonstructural proteins (2A–2C, 3A–3D) [9,10]. 2A and 3C are viral cysteine proteases (2A^pro^ and 3C^pro^) that cleave viral polyproteins co-translationally and post-translationally to produce mature viral proteins [11,12]. 3D^pol^ is the RNA-dependent RNA polymerase (RdRp) required for virus genome replication [1,13]. The pathogenesis of CVB infection is largely unknown. Evidence has shown that multiple cellular processes, such as the ubiquitin-proteasome system and autophagy, are manipulated by CVB to facilitate viral replication [14,15,16]. CVB promotes the formation of autophagosomes, which serve as viral replication membranes [17]. Rapamycin has also been shown to stimulate autophagosome formation, thereby facilitating CVB replication [18,19]. Autophagy inhibitors, on the other hand, inhibit CVB replication [18,20].

It has been demonstrated that CVB subverts autophagy to facilitate viral replication [15]. Autophagy is a highly conserved pathway for degradation by which non-functional organelles and macromolecules are delivered to lysosomes, where they are degraded. Autophagy also eliminates invasive pathogens [21,22]. Autophagy is composed of macroautophagy (hereafter referred to as autophagy), microautophagy, and chaperone-mediated autophagy [23]. Autophagy is a highly regulated process starting with autophagosome formation, which receives lysosomal enzymes by fusing with transport vesicles from the trans-Golgi network or lysosomes, where cellular constituents are degraded and recycled [24]. During the formation of autophagosomes, microtubule-associated protein light chain 3 (LC3) is inserted into the autophagosome membrane through lipidation, in which the cytosolic LC3 I is converted to membrane-bound LC3-II [23]. Cargo selection for lysosomal degradation is mediated by autophagic adaptor proteins such as p62 and NBR1 [25,26]. Autophagic adaptors, which interact with both cargos and LC3-II, guide the incorporation of cargos into autophagosomes and are degraded with cargos in lysosomes [23,27]. Therefore, the dynamic process of autophagy, referred to as autophagic flux, is represented by the progressive degradation of LC3-II and p62 determined by immunoblotting [22].

N-acetylcysteine (NAC) is a chemical that contains thiols and serves as a precursor to glutathione and L-cysteine [28]. It is a safe and well-tolerated medication widely used in clinical practice [29]. NAC also has antiviral effects against influenza [30] and HIV [31]. It was proposed that NAC could be an antiviral option for managing COVID-19 patients [32]. Studies also showed that NAC exhibited a cardioprotective effect by inhibiting excessive autophagy [33] and pyroptosis [34] induced by ischemia-reperfusion. Our previous study shows that NAC inhibits CVB replication by inhibiting the activation of caspase-1, which facilitates CVB replication by an unknown mechanism [16]. Considering the potent antiviral effect of NAC and its multiple functional mechanisms, we supposed that the molecular mechanism underlying the anti-CVB effect of NAC deserves further study.

In this study, we investigated how NAC exerts antiviral effects against CVB infection. We showed that NAC treatment led to reduced expression and upregulated degradation of eukaryotic elongation factor 1 alpha 1 (eEF1A1), which was required for CVB3 replication. We also identified that the autophagic degradation of eEF1A1 was mediated by p62/SQSTM1.

## 2. Materials and Methods

### 2.1. Ethnics Statement

The use of laboratory animals in this study was approved by the Ethics Committee of Harbin Medical University. The experiment procedures followed the guidelines for humane animal treatment from the Ethics Committee of Harbin Medical University.

### 2.2. Mice

Newborn Balb/c mice were purchased from the Laboratory Animal Center of Harbin Medical University (Harbin, China). Mice were kept in a pathogen-free facility at 24 °C and 45% humidity and allowed to access water and food ad libitum. Mice were infected with CVB3 five days after birth by peritoneal injection. Sham-infected mice were given the same amount of Dulbecco’s Modified Eagle Medium (DMEM) (Thermo Fisher, Shanghai, China).

### 2.3. Cell Culture

HeLa cells were cultured in DMEM (Thermo Fisher) supplemented with 10% fetal bovine serum (FBS, Bioindustry, Israel), penicillin (100 U/L), and streptomycin (100 U/L). Cells were incubated in 5% CO_2_ at 37 °C and passaged every two days.

### 2.4. Virus

CVB3 woodruff was kindly provided by the Scrips Institute (San Diego, CA, USA). The virus stock solution was stored at −80 °C, and virus titer was determined by 50% tissue culture infectious dose (TCID_50_) as described previously [35]. Briefly, cells were infected with the virus for 24 h. Cell culture was harvested and freeze-thawed three times. Virus preparation was obtained by the centrifugation of the freeze-thawed culture harvest for 5 min at 1000 rpm. HeLa cells were infected with virus preparation at a series of dilutions. Cytopathic effect (CPE) was calculated 24 h p.i.

### 2.5. Virus Absorption

HeLa cells were added to the CVB3 solution with or without NAC and cultured at 4 °C to permit virus absorption. After incubating for 1 h, the cells were washed with cold PBS twice to stop absorption.

### 2.6. Virus Entry

HeLa cells were added to the CVB3 solution with or without NAC and cultured at 4 °C to absorb CVB3. After incubation for 1 h, the supernatant was removed, and the cells were washed with cold PBS twice to remove the unbound virus. Then, the fresh culture medium was added to the cells cultured at 37 °C for 1 h to allow the virus to enter the cells.

### 2.7. Transfection

HeLa cells were cultured to 70% of confluence in 6-well plates. The transfection mix was prepared with 4 μg plasmid or 25 nmol siRNA (RiboBio, Guangzhou, China) and 3.75 μL of Lipofectamine 3000 (Thermo Fisher) in 1000 μL of DMEM. The culture medium was removed, and cells were covered with the transfection mix and incubated for 4 h at 37 °C. The transfection mix was removed and replaced with a fresh culture medium. Cells were harvested at 24 or 48 h after transfection for RT-qPCR and immunoblotting.

### 2.8. RT-qPCR

Total RNA was extracted by TRIzol (Invitrogen) according to the instructions recommended by the manufacturer. RNA was dissolved in nuclease-free water and was quantified by Nanodrop 2000 (Thermo Fisher, Waltham, MA, USA). An amount of 20 μL of reverse transcription system was prepared with 1 μg of RNA and 4 μL of 5× Trans Script All-in-One Super Mix (Trans Gen, Beijing, China). Reverse transcription was carried out by incubating the transcription mix at 42 °C for 15 min, followed by heating at 85 °C for 15 s. Quantitative PCR was carried out on Light Cycler 96 (Roche, Basel, Switzerland) with Trans Start Top Green qPCR Super Mix (Trans Gen, Beijing, China). The PCR system consisted of 1 μL of cDNA, 0.4 μL of each primer (10 μM), and 10 μL of 2× Trans Start Top Green qPCR Super Mix. The PCR was carried out for 45 cycles, and each cycle consisted of denaturation at 94 °C for 5 s, annealing at 58 °C for 15 s, and extension at 72 °C for 1 min. GAPDH was used as the internal control to normalize RNAs. The PCR products were calculated by the 2^−ΔΔCt^ method. Primers were synthesized by Comate Bio (Jilin, China). The sequences of the primers are listed as follows: CVB3 forward primer 5′-GCACACACCCTCAAACCAGA-3′ and reverse primer 5′-ATGAAACACGGACACCCAAAG-3′; GAPDH forward primer 5′-GGAGCGAGATCCCTCCAAAAT-3′ and reverse primer 5′-GGCTGTTGTCATACTTCTCATGG-3′; GAPDH (mice) forward primer 5′-AGGTCGGTGTGAACGGATTTG-3′ and reverse primer 5′-GGGGTCGTTGATGGCAACA-3′; EEF1A1 forward primer 5′-TGTCGTCATTGGACACGTAGA-3′ and reverse primer 5′-ACGCTCAGCTTTCAGTTTATCC-3′; EEF1A1 (mice) forward primer 5′-ACACGTAGATTCCGGCAAGTC-3′ and reverse primer 5′-GATGGTTCGCTTGTCGATTCC-3′; EEF1A2 forward primer 5′-GTCAAGGAAGTCAGCGCCTAC-3′ and reverse primer 5′-TGAACCACGGCATGTTGGG-3′; EEF1A2 (mice) forward primer 5′-ACTCCACGGAACCAGCCTA-3′ and reverse primer 5′-GGGCAGGATTGTGTCCAGG-3′; NDRG1 forward primer 5′-CTCCTGCAAGAGTTTGATGTCC-3′ and reverse primer 5′-TCATGCCGATGTCATGGTAGG-3′; MT-CYB forward primer 5′-TCTTGCACGAAACGGGATCA-3′ and reverse primer 5′-GTGGGGAGGGGTGTTTAAGG-3′; ALPI forward primer 5′-TGAGGGTGTGGCTTACCAG-3′ and reverse primer 5′-GATGGACGTGTAGGCTTTGCT-3′; HSPA5 forward primer 5′-CACGGTCTTTGACGCCAAG-3′ and reverse primer 5′-CCAAATAAGCCTCAGCGGTTT-3′; and HIST2H2AA3 forward primer 5′-ATCATCCCTCGTCACCTCCA-3′ and reverse primer 5′-CCTTGTGGTGACTCTCCGTC-3′.

### 2.9. RNA-Sequencing

HeLa cell were cultured in DMEM supplemented with 2% FBS to 70% confluency and infected with CVB3 at an MOI of 1 for 24 h with or without NAC treatment. Total RNA was extracted with TRIzol following the instructions of the manufacturer. RNA quantity and quality were assessed using a NanoDrop2000 spectrophotometer (Thermo Fisher, Waltham, MA, USA) and subjected to second generation sequencing by GeneX Health (Beijing, China). The data of the gene expression profile were analyzed with Graphpad Prism 9 and presented as a volcano plot.

### 2.10. Western Blot

Cells cultured in 6-well plates were harvested by the removal of the culture medium and washed with cold PBS twice. A total of 100 μL of RIPA buffer (Beyotime, Wuhan, China) containing 1% protease inhibitor PMSF (Beyotime) was added to collect the cells. Cells were lysed for 20 min on ice, followed by centrifugation at 12,000 rpm at 4 °C for 15 min to collect the supernatant. The supernatant was stored at −80 °C. The protein concentration was measured using a BCA protein assay kit (Beyotime). A total of 10% SDA-PAGE gel was prepared to separate protein samples. After electrophoresis, proteins were transferred to polyvinylidene difluoride (PVDF) membranes (Millipore, Kenilworth, NJ, USA). PVDF membranes were blocked with skimmed milk for 1 h and incubated with the primary antibody for 2 h at room temperature. Membranes were washed with 0.1% Tween-20 in TBST and incubated with anti-rabbit or anti-mouse IgG for 1 h at room temperature. Finally, the membrane was washed, rinsed with ECL detection reagent (Beyotime), and visualized by Tanon 5200 Chemi-Image System (Biotanon, Shanghai, China).

### 2.11. Co-Immunoprecipitation

A 500 μL protein sample was mixed with antibody conjugated on immunomagnetic beads and incubated at room temperature for 1 h on a rotating mixer. Then, the mixture was placed on a magnetic stand for 1 min to remove the supernatant. The mixture was washed by gentle pipetting with 500 μL rinsing buffer (Epizyme) 10 times. Finally, 100 μL of elution buffer was added to the IP mixture and incubated for 10 min at room temperature. The supernatant was collected for immunoblotting.

### 2.12. NAC Preparation and Treatment

NAC (Sigma Aldrich, St. Louis, MO, USA) was dissolved in PBS. The NAC solution was aliquoted and stored at −80 °C. The working solution of NAC was used only once to avoid oxidation. For in vivo experiments, mice that were infected with CVB3 (10^6^ TCID_50_) were given 15 mg/kg (body weight) of NAC intraperitoneally twice a day for five consecutive days, starting 12 h p.i. Mice were euthanized at the end of day 5 of infection.

### 2.13. Cycloheximide Chase Assay

Cycloheximide (CHX) (Abmole Shanghai, China) was dissolved in DMSO to prepare the stock solution (20 mg/mL), which was stored at −20 °C. HeLa cells were used to carry out the CHX chase assay. Cells were treated with CHX at a working concentration (20 μg/mL) at different timepoints p.i. Cell lysates were prepared and eEF1A1 and LC3 were determined by immunoblotting.

### 2.14. Cell Viability

HeLa cells were cultured in 96-well plates and exposed to various concentrations of NAC for 24 h. The Cell Counting Kit-8 (CCK-8) (Beyotime, Wuhan, China) was used to measure cell viability following the protocol recommended by the provider. To determine cell viability, the culture medium was removed. Cells were covered with CCK-8 reagent (5 mg/mL) and incubated for 2 h to allow the formation of formazan crystals. Cells were washed three times with PBS, followed by the addition of 150 μL DMSO to dissolve the formazan crystals. Cell viability was determined at 570 nm by the microplate reader Epoch2 (BioTek). The 50% cytotoxic concentration (CC_50_) was designated as the concentration at which NAC reduced cell viability by precisely 50%.

### 2.15. Histological Examination

Mouse hearts were fixed and embedded in paraffin. Cardiac tissues were sectioned and underwent HE staining. The tissue sections were examined separately by two specialists from the Department of Pathology of Harbin Medical University.

### 2.16. Statistical Analysis

All of the experiments were repeated at least three times. The quantitative data were analyzed by Graphpad Prism 9 and presented as mean ± SD. The Student’s *t* test and one-way ANOVA were used for statistical analysis. A *p*-value less than 0.05 is considered statistically significant. Data were analyzed by GraphPad Prism 9.

## 3. Results

### 3.1. NAC Inhibits CVB3 Replication

**Figure 1:**
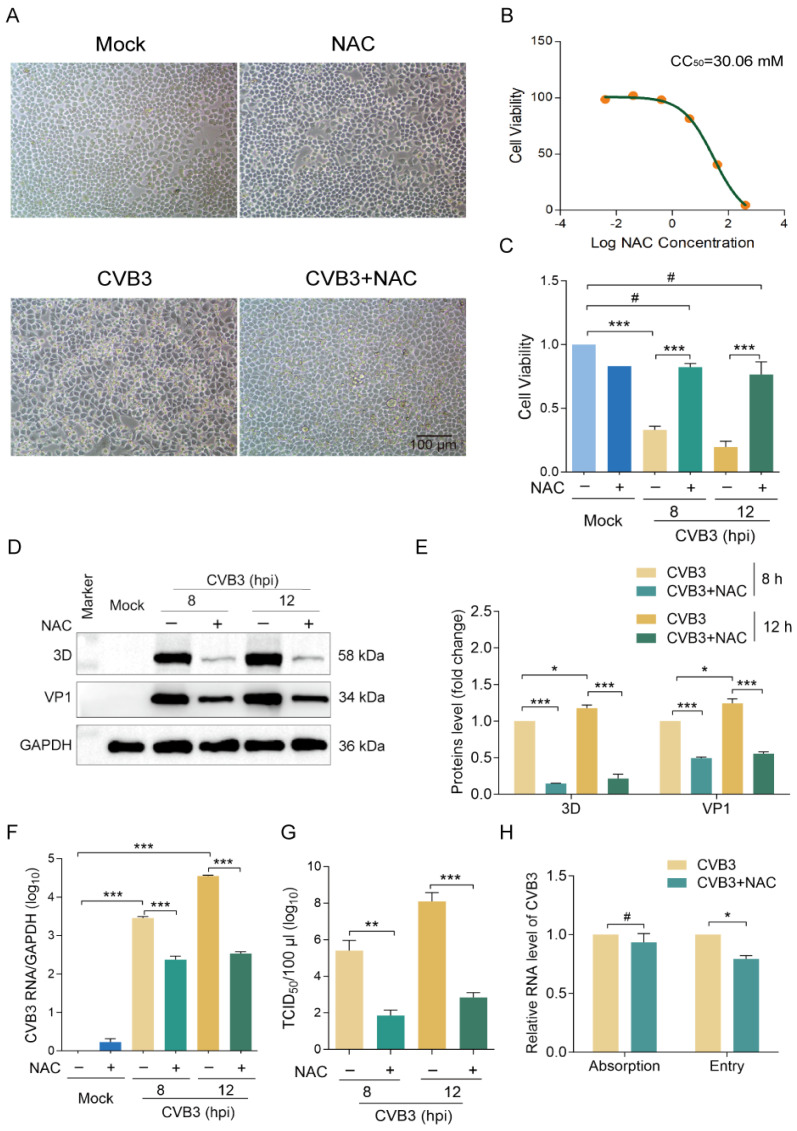
NAC inhibits CVB3 replication and reduces CPE. (**A**) HeLa cells were infected with CVB3 (MOI = 10) for 8 h in the medium supplemented with NAC (20 mmol/L). The cytopathic effect was observed in microscopy. (**B**) HeLa cells were treated with NAC at various concentrations for 24 h. The CC_50_ of NAC was measured. (**C**) HeLa cells were infected with CVB3 (MOI = 10) and treated with NAC for 8 h or 12 h. Cell viability was determined. (**D**–**G**) HeLa cells were infected with CVB3 (MOI = 10) and treated with NAC for 8 h or 12 h. Cells were collected and subjected to immunoblotting (**D**,**E**), RT-qPCR (**F**), and TCID_50_ assay (**G**). (**H**) HeLa cells were infected with CVB3 (MOI = 10) in the medium supplemented with or without NAC (20 mmol/L). Virus adsorption and virus entry were determined as described in detail in Materials and Methods. Cells were collected, and viral RNA was determined by RT-qPCR. Experiments were repeated at least three times. * *p* < 0.05; ** *p* < 0.01; *** *p* < 0.001; # no significance. hpi: hours of post-infection.

First, we evaluated the antiviral effect of NAC against CVB in vitro. HeLa cells were infected with CVB3 at an MOI of 10 and treated with 20 mmol/L NAC. Cells were cultured for 8 h or 12 h, and cell viability was determined. We show that NAC significantly inhibited the cytopathic effect and increased cell survival (Figure 1A–C). Accordingly, viral RNA, viral protein VP1 and 3D^pol^, and viral particles were significantly reduced in the cells treated with NAC treatment (Figure 1D–G), demonstrating that NAC effectively inhibited CVB3 replication. Virus infection begins with the absorption and the penetration of viruses to the plasma membrane. To estimate how NAC affects the virus life cycle, we next determined if NAC affects virus adsorption and entry into the cells. We show that NAC did not affect virus adsorption (Figure 1H), while the viruses which entered into the cells were slightly but significantly reduced due to NAC treatment.

### 3.2. NAC Inhibits the Expression of eEF1A1, Which Is Upregulated by CVB3 Infection

**Figure 2:**
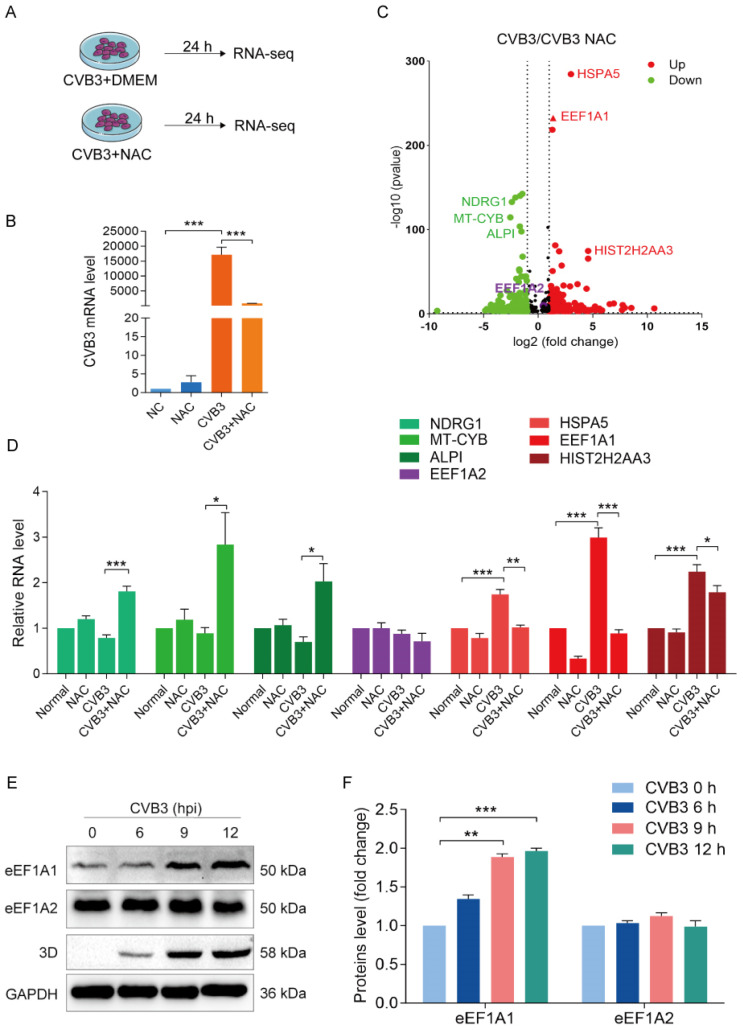
NAC inhibits the expression of eEF1A, which is upregulated by CVB3 infection. (**A**–**C**) The gene expression profile was determined in CVB3-infected cells with or without NAC treatment. HeLa cells were infected with CVB3 (MOI = 1) in the medium supplemented with NAC (20 mmol/L) for 24 h (**A**). Viral replication was confirmed by determining CVB3 genomic RNA with RT-qPCR (**B**). Total RNA was extracted and subjected to the analysis of RNA-seq. The gene expression profile of CVB3-infected cells with or without NAC treatment was presented in the volcano plot (**C**). (**D**) The expression of representative genes identified by RNA-seq was validated by RT-qPCR. HeLa cells were infected with CVB3 (MOI = 1) for 24 h. Total RNA was extracted and subjected to RT-qPCR. (**E**,**F**) The abundance of the protein levels of eEF1A1 and eEF1A2 was determined in CVB3-infected cells. Cells were infected with CVB3 (MOI = 1). Cell lysates were harvested at different timepoints of p.i. and analyzed by immunoblotting. *n* = 3. * *p* < 0.05, ** *p* < 0.01; *** *p* < 0.001.

Although virus entry was slightly inhibited in CVB3-infected cells with NAC treatment, the dramatically decreased virus yield suggests that other mechanisms are involved. To identify the antiviral mechanisms of NAC, the gene expression profile of CVB3-infected cells with or without NAC treatment was determined by RNA-sequencing (RNA-seq) (Supplementary file and Figure 2A–C). We analyzed the RNA-seq data and identified that *EEF1A1*, but not *EEF1A2,* which is highly homologous to *EEF1A1*, was one of the highly upregulated genes in CVB3-infected cells (Figure 2C). To validate the gene expression profile during CVB3 infection with or without NAC treatment, the expression levels of several genes that are dramatically altered as identified by RNA-seq were determined by RT-qPCR (Figure 2D). In agreement with the data of RNA-seq (Figure 2C), the expression of *NDRG1*, *MT-CYB*, and *ALPI* was not altered in CVB3-infected cells, while NAC treatment upregulated the expression of these genes (Figure 2D). Also in agreement with the data of RNA-seq, the expression levels of *HSPA5*, *EEF1A1*, and *HIST2H2AA3* were significantly upregulated in CVB3-infected cells, while NAC treatment suppressed the expression of these genes (Figure 2D). Moreover, *EEF1A2* expression remained unchanged in CVB3-infected cells regardless of NAC treatment (Figure 2D). These data suggest that the antiviral mechanism of NAC might be related to its inhibition of eEF1A1 expression.

eEF1A1 is the subunit of the eukaryotic translation elongation 1 complex [36]. In addition to its canonical role in translation elongation, eEF1A1 exerts diverse functions in multiple cellular processes such as cell proliferation, apoptosis, and cytoskeleton organization [37]. Evidence has shown that eEF1A1 facilitates the replication of hepatitis delta virus (HDV) by binding to viral genomes [38]. Importantly, our previous study demonstrated that eEF1A1 was required for CVB3 replication [39]. Considering that eEF1A1 expression was upregulated in response to CVB3 infection and suppressed by NAC treatment, we hypothesized that the antiviral mechanism of NAC is associated with its inhibitory effect on eEF1A1. We then reconfirmed the expression of eEF1A1 in CVB3-infected cells and found that the abundance eEF1A1 protein was significantly upregulated in response to CVB3 infection in a time-dependent manner, while eEF1A2 was unchanged (Figure 2E,F). These data show that CVB3 infection increased eEF1A1 expression, and NAC suppressed eEF1A1 expression.

### 3.3. CVB3 Replication Depends on eEF1A1

**Figure 3:**
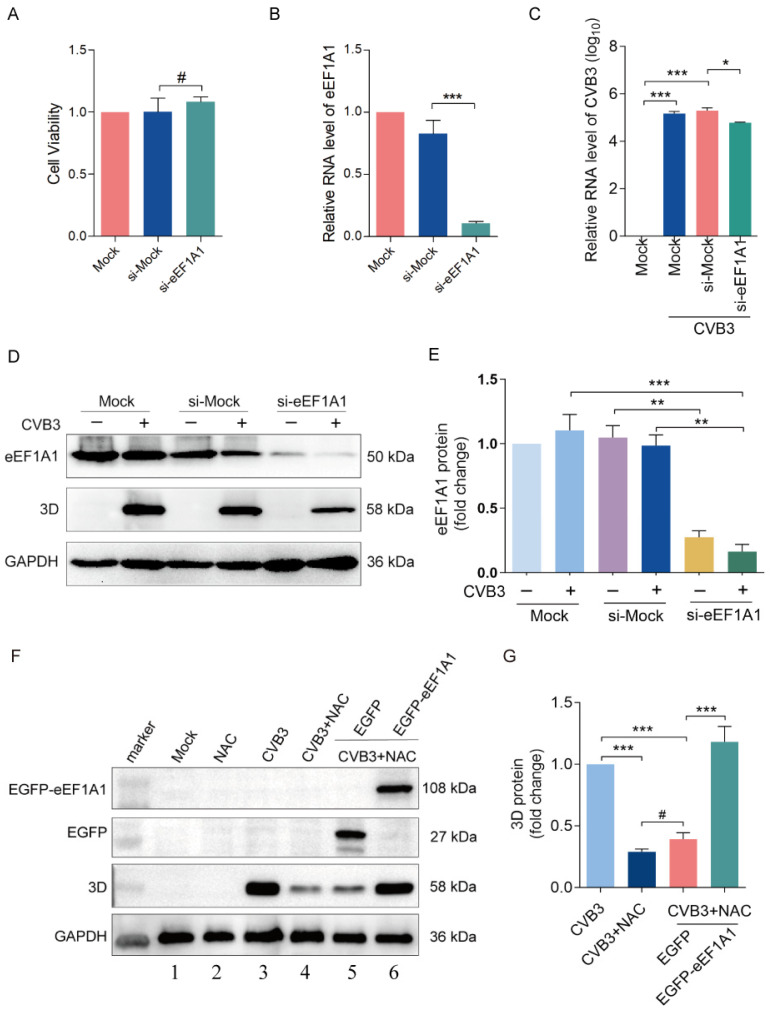
CVB3 replication depends on eEF1A1. (**A**–**E**) HeLa cells were transfected with siRNA of eEF1A1 for 12 h and then infected with CVB3 for 8 h. (**A**) Cell viability was determined. (**B**,**C**) Total RNA was extracted and subjected to RT-qPCR. (**D**,**E**) Cell lysates were collected and subjected to immunoblotting. (**F**,**G**) HeLa cells were transfected with EGFP-eEF1A1 for 12 h and then infected with CVB3 for 12 h in the medium supplemented with NAC. Cell lysates were collected and subjected to immunoblotting. *n* = 3. * *p* < 0.05; ** *p* < 0.01; *** *p* < 0.001; # no significance.

To show the correlation between eEF1A1 and CVB3 infection, we determined the impact of eEF1A1 knockdown on viral replication. Since eEF1A1 plays an essential role in translation elongation, the application of the siRNA of eEF1A1 was carefully controlled in order to maintain cell viability (Figure 3A), while at the same time, the knockdown effect was reached (Figure 3B). We show that the depletion of eEF1A1 significantly inhibited CVB3 replication, as represented by the reduced viral RNA level (Figure 3C) and 3D^pol^ (Figure 3D,E). To further estimate the correlation between CVB3 replication and eEF1A1, viral replication was determined in the cells with eEF1A1 overexpression. To this end, HeLa cells were transfected with pEGFP-eEF1A1 for 24 h, followed by the infection of CVB3 (MOI = 10) with or without NAC treatment. We show that eEF1A1 overexpression partially but significantly reversed the viral 3D^pol^ level, which was downregulated by NAC treatment (Figure 3F: lane 6 vs. lane 5; Figure 3G). These results indicate that CVB3 replication at least partially depends on eEF1A1.

### 3.4. NAC Alleviates Myocarditis and Downregulates eEF1A1 in the Myocardium Infected with CVB3

**Figure 4:**
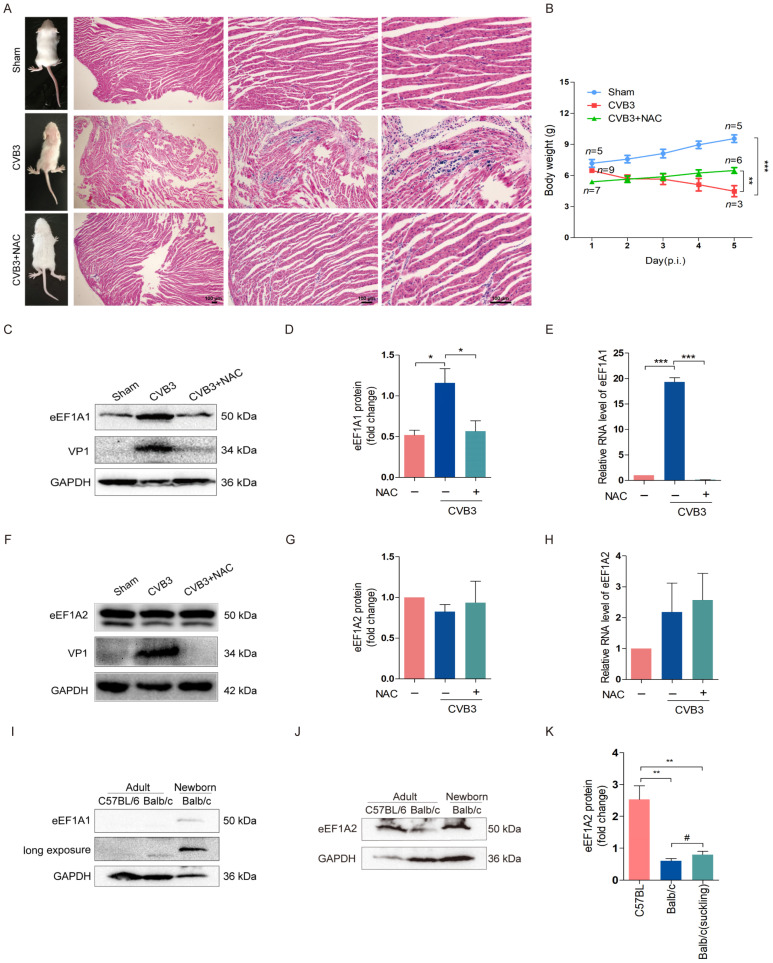
NAC alleviates myocarditis and downregulates eEF1A1 in the myocardium infected with CVB3. (**A**,**B**) Newborn Balb/c mice were infected with CVB3 at 10^6^ TCID_50_. Mice were treated with NAC (15 mg/kg body weight) by intraperitoneal injection at 12 h p.i. twice a day for five consecutive days. The overall condition of the mice was shown at the end of day 5 p.i. (**A**). Mouse hearts were collected and subjected to histological study by HE staining (**A**). The body weights of the mice were monitored per day and the survived mice were recorded at day 5 of p.i. (**B**). *n* = 5–9. (**C**–**H**) Mice were treated as described in A and B. Total proteins and RNA were extracted from the myocardium and subjected to immunoblotting (**C**,**D**,**F**,**G**) or RT-qPCR (**E**,**H**). (**I**–**K**) Myocardium was collected from the hearts of C57BL/6 and Balb/c mice and subjected to the analysis of immunoblotting to determine eEF1A1 (I) or eEF1A2 (**J**,**K**). Newborn Balb/c mice were at the age of 5 to 7 days after birth. *n* = 3. * *p* < 0.05; ** *p* < 0.01; *** *p* < 0.001; # no significance.

Next, the antiviral effect of NAC was further confirmed in CVB3-infected mice, and eEF1A1 expression in the mouse myocardium was determined to show the impact of NAC. Newborn Balb/c mice were infected with 10^6^ TCID_50_ of CVB3 by intraperitoneal injection. Mice were treated with NAC (15 mg/kg body weight) intraperitoneally twice a day for five consecutive days starting 12 h after viral infection. On day 5 post-infection (p.i.), mice were euthanized, and the myocardium was subjected to histological examination. As shown in Figure 4, the limb paralysis and weight loss of mice infected with CVB3 were obvious, while mice treated with NAC exhibited a dramatically improved overall condition and body weight gain (Figure 4A,B). At day 5 post-infection, most mice (6/7) treated with NAC were alive, while 2/3 (6/9) of CVB3-infected mice without NAC treatment were dead (Figure 4A,B). The myocardial injury was obvious for CVB3-infected mice with damaged myofibrils and inflammatory infiltration (Figure 4A). In contrast, NAC treatment significantly improved the integrity of myofibrils and dramatically reduced the inflammatory foci (Figure 4A).

To study the correlation between CVB infection and eEF1A, we determined the expression of eEF1A (eEF1A1 and eEF1A2) and viral proteins VP1 in the myocardia of the mice infected with CVB3. We show that CVB3 infection significantly upregulated the eEF1A1 level in the myocardium, which was suppressed by NAC treatment (Figure 4C–E). In contrast, eEF1A2 expression remained unchanged in CVB3-infected mice with or without NAC treatment (Figure 4F–H).

The differential expression of eEF1A1 and eEF1A2 in the myocardia of adult and newborn mice was also determined (Figure 4I–K). Previous studies demonstrated that eEF1A2 is ubiquitously expressed during embryonic development and after birth, while eEF1A1 is expressed primarily in embryos and specific tissues of adult mammals such as the myocardium and neural system [40,41]. In agreement with previous reports, eEF1A1 was absent in the adult mouse myocardium of both C57BL/6 and Balb/c mice (Figure 4C), while eEF1A2 was expressed by both species (Figure 4I). The newborn Balb/c mice expressed eEF1A1 and eEF1A2 (Figure 4J,K). These results show that eEF1A1, which was upregulated in response to CVB3 infection in the myocardia of newborn mice, was dramatically decreased by NAC treatment.

### 3.5. NAC Promotes eEF1A1 Degradation

**Figure 5:**
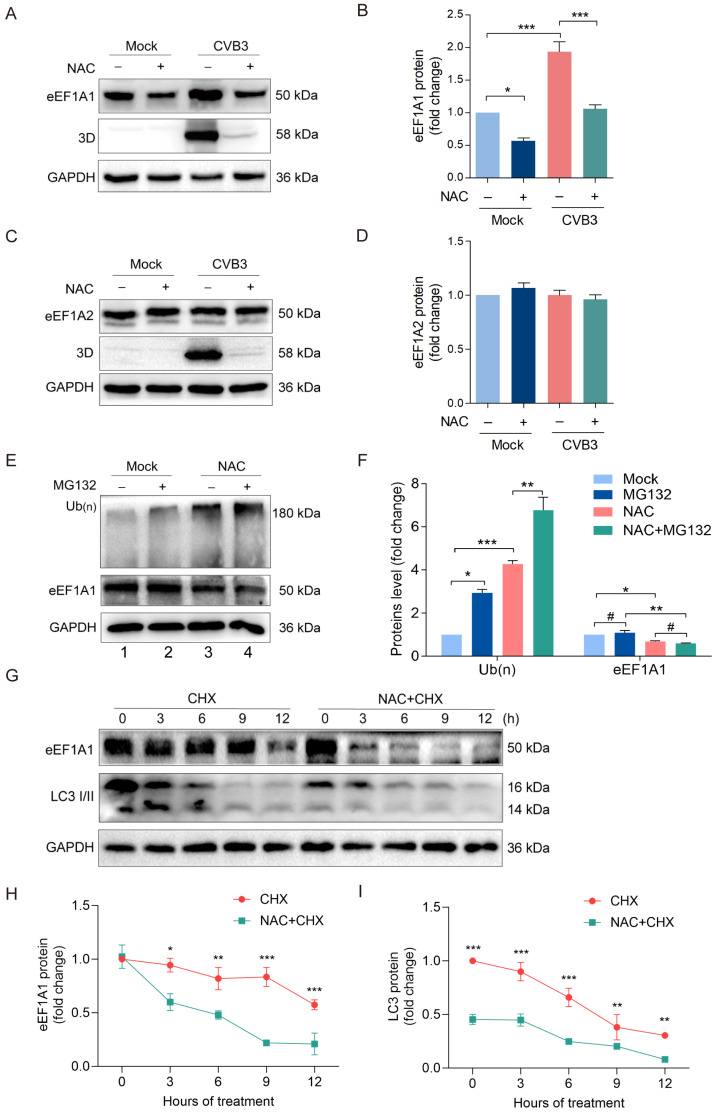
NAC promotes eEF1A1 degradation. (**A**–**D**) HeLa cells were infected with CVB3 (MOI = 1) for 12 h in the medium supplemented with NAC (20 mmol/L). Cell lysates were prepared and analyzed by immunoblotting. (**E**,**F**) HeLa cells were treated with MG132 or MG132 in combination with NAC. Total cellular proteins were collected to analyze by immunoblotting. (**G**–**I**) HeLa cells were treated with CHX or CHX in combination with NAC. Total cellular proteins were collected at different timepoints after CHX treatment and analyzed by immunoblotting. *n* = 4. * *p* < 0.05; ** *p* < 0.01; *** *p* < 0.001; # no significance.

Thus far, our data suggest that the antiviral mechanism of NAC against CVB3 infection might be related to the downregulated eEF1A1. It has been shown that autophagy was significantly enhanced in a time-dependent manner in NAC-treated yeast cells [42]. Our recent work demonstrated that eEF1A1 was degraded by chaperone-mediated autophagy through interaction with heat shock cognate protein 70 (Hsc70) [39]. Here, we show that NAC downregulated eEF1A1 at both mRNA (Figure 2B) and protein levels (Figure 4C). However, it is unclear whether the downregulated eEF1A1 results from transcriptional inhibition alone or in combination with the enhanced protein degradation of eEF1A1. To this end, the inhibitory effect of NAC on eEF1A1 expression was confirmed again in CVB3-infected cells (Figure 5A,B). We show that NAC treatment moderately but significantly reduced the eEF1A1 protein level, while eEF1A2 was not altered (Figure 5C,D). Next, we investigated if the ubiquitin–proteasome system (UPS) is involved in the reduction in eEF1A1 induced by NAC treatment, since UPS is the major protein degradation machinery which primarily eliminates soluble cytoplasmic proteins [43]. To this end, HeLa cells were treated with NAC for 12 h, followed by the treatment of MG132 for 6 h. Cells were collected to determine ubiquitinated proteins and eEF1A1 (Figure 5E,F). We show that MG132 treatment led to the accumulation of ubiquitinated proteins, which was further enhanced by the treatment of NAC (Figure 5E: lane 4 vs. lane 2; Figure 5F). Moreover, NAC treatment alone obviously promoted the accumulation of the ubiquitinated proteins (Figure 5E: lane 3 vs. lane 1; Figure 5F). These results suggest that NAC either upregulates protein ubiquitination or blocks the degradation of the ubiquitinated proteins. In the case of eEF1A1, its protein level was not changed in the cells treated with MG132 (Figure 5E: lane 2 vs. lane 1; Figure 5F). In contrast, NAC treatment significantly downregulated the eEF1A1 level (Figure 5E: lane 3 vs. lane 1; Figure 5F) even in the cells treated with both NAC and MG132 (Figure 5E: lane 4), while ubiquitinated proteins were obviously accumulated. These observations indicate that NAC downregulates the protein level of eEF1A1 in proteasome-independent manner.

Subsequently, we determined the role played by autophagy in the regulation of eEF1A1. To this end, the cycloheximide (CHX) chase assay was used to determine the degradation of eEF1A1 and LC3. HeLa cells were treated with CHX or CHX in combination with NAC, and cells were collected to determine eEF1A1 and LC3I/II at various time points (Figure 5G–I). We found that eEF1A1 remained at relatively high level 9 h after CHX treatment, while it was almost undetectable at 9 h after the treatment with both CHX and NAC (Figure 5G,H), demonstrating that NAC accelerated eEF1A1 degradation. Moreover, NAC also accelerated LC3 degradation (Figure 5G,I), indicating that NAC promotes autophagic flux since LC3 II itself is degraded in lysosomes along with autophagosomes. Taken together, these data indicate that NAC enhances eEF1A1 degradation by upregulating autophagy.

### 3.6. NAC Enhances eEF1A1 Degradation by Promoting Autophagy during CVB3 Infection

**Figure 6:**
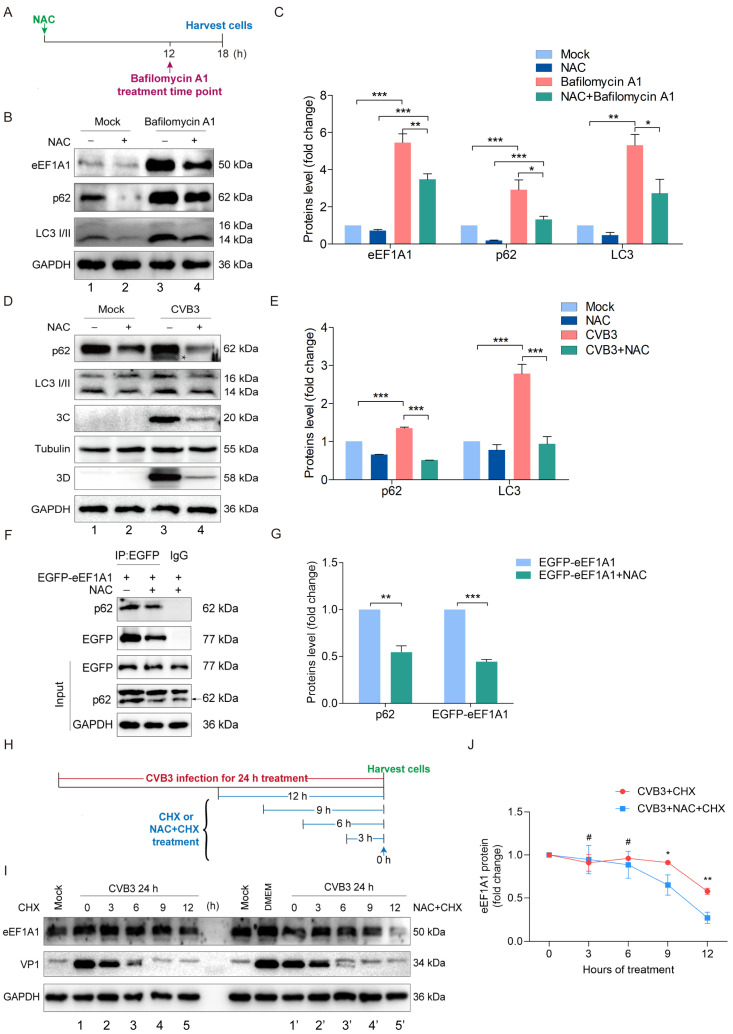
NAC enhances eEF1A1 degradation by promoting autophagy during CVB3 infection. (**A**–**C**) Cells were treated with NAC (20 mmol/L) for 12 h followed by the treatment of bafilomycin A1 for 6 h (**A**). Cells were harvested and subjected to immunoblotting (**B**,**C**). (**D**,**E**) HeLa cells were infected or mock-infected with CVB3 (MOI = 1) for 24 h in the medium with or without NAC (20 mmol/L) supplementation. Cells were harvested and analyzed by immunoblotting. (**F**,**G**) HeLa cells were transfected with pEGFP-eEF1A1 for 12 h and then treated with NAC for 12 h. Cell lysates were prepared and subjected to IP with anti-EGFP antibody. Immunoblotting was performed with anti-p62 and anti-EGFP antibodies. (**H**−**J**) HeLa cells were infected with CVB3 for 12 h followed by the treatment of CHX or the combination of CHX and NAC at different timepoints of p.i. Cells were harvested and analyzed by immunoblotting. hpi: hours post-infection. * *p* < 0.05; ** *p* < 0.01; *** *p* < 0.001; # no significance.

Thus far, we have shown that NAC enhanced eEF1A1 degradation by promoting autophagy in uninfected cells (Figure 5G–I). To support the hypothesis that the upregulated eEF1A1 degradation induced by NAC was due to the enhanced autophagy, eEF1A1 was determined in the cells treated with either NAC or the combination of NAC and bafilomycin A1 (Figure 6A–C). To this end, HeLa cells were treated with NAC for 12 h, followed by the treatment of bafilomycin A1 for an additional 6 h, which inhibits autophagic flux by blocking the acidification of lysosomes (Figure 6A,B). We show that the protein levels of eEF1A1, LC3 II, and p62 were dramatically increased in the cells treated with bafilomycin A1 (Figure 6B: lane 3 vs. lane 1) due to the inhibited lysosomal degradation. In contrast, the addition of NAC downregulated the accumulation of eEF1A1, LC3 II, and p62 (Figure 6B: lane 4 vs lane 3; Figure 6C) in the presence of bafilomycin A1, indicating that NAC partially overcame the blocked autophagy pathway. NAC treatment alone obviously downregulated p62 and LC3 II (Figure 6B: lane 2 vs. lane 1). In NAC-treated cells, the addition of bafilomycin resulted in the significant accumulation of eEF1A1, LC3 II, and p62 (Figure 6B: lane 4 vs. lane 2; Figure 6C), indicating that, similar to LC3 II and p62, eEF1A1 is degraded through autophagy.

To show how NAC would impact autophagy during CVB3 infection, cells were infected with CVB3 (MOI = 10) for 12 h in the presence or absence of NAC. p62 and LC3 I/II were determined to show the autophagic flux. In agreement with the results above (Figure 6B,C), NAC treatment reduced both p62 and LC3 II moderately (Figure 6D: lane 2 vs. lane 1; Figure 6E), while CVB3 infection resulted in a moderate increase in both the p62 and LC3 II level (Figure 6D: lane 3 vs. lane 1; Figure 6E), due to the enhanced formation of autophagosomes and the blocked autophagic flux [44]. Compared to the accumulation of autophagic adaptor proteins p62 and LC3 II in CVB3-infected cells (Figure 6D: lane 3; Figure 6E), which is the result of the inhibited degradation of autophagosomes, NAC treatment led to a dramatic reduction in both p62 and LC3 I/II (Figure 6D: lane 4; Figure 6E).

We also noted the cleavage of p62 in CVB3-infected cells (Figure 6D: lane 3, indicated by asterisk), demonstrating the production of viral 3C^pro^ along with CVB3 replication (Figure 6D: lane 3). In contrast, NAC treatment led to a marked decrease in both p62 and LC3 (Figure 6: lane 4). Moreover, both viral 3C^pro^ and 3D were significantly reduced (Figure 6D: lane 4; Figure 6E), demonstrating the suppressed viral replication. These data also indicate that the reduced p62 in CVB3-infected cells with NAC treatment was not the result of the increased cleavage of p62, since the production of viral 3C^pro^, the protease that cleaves p62 and other cellular proteins, was downregulated. In other words, the dramatic reduction in p62 in CVB3-infected cells with NAC treatment was the result of inhibited viral replication.

The blocked autophagic flux was well documented by CVB3 infection, as shown by the accumulation of p62 and LC3II (Figure 6D: lane 3). Therefore, our data also suggest that NAC treatment promotes the autophagic degradation of p62 in CVB3-infected cells, since NAC blocks the degradation of ubiquitinated protein and consequently inhibits the proteasome (Figure 5E).

For the autophagic degradation of proteins or organelles, p62 plays a central role as the adaptor protein for cargo selection [45]. Therefore, we proposed that autophagic degradation of eEF1A1 depends on its interaction with p62. To this end, the interaction between eEF1A1 and p62 was determined by immunoprecipitation in the presence or absence of NAC (Figure 6F,G). In order to maintain protein level, eEF1A1 overexpression was performed for 24 h before the treatment of NAC, since NAC promotes the degradation of both eEF1A1 and p62 (Figure 6B). The interaction between eEF1A1 and p62 was demonstrated in the cells with or without NAC treatment (Figure 6F), demonstrating that eEF1A1 intrinsically binds to p62. NAC treatment led to an obvious decrease in both eEF1A1 and p62, which were bound to one another (Figure 6F,G), suggesting that NAC enhances the autophagic degradation of eEF1A1.

To further verify that NAC enhances eEF1A1 degradation during CVB3 infection, the CHX chase assay was used to determine the degradation rate of eEF1A1. To this end, HeLa cells were infected with CVB3 (MOI = 10) for 12 h, followed by the treatment of CHX in combination with or without NAC for various time durations (Figure 6H). We show that the eEF1A1 level was almost undetectable when cells were treated with CHX and NAC for 12 h (Figure 6H,I), while the eEF1A1 level remained moderate in the cells treated with CHX alone (Figure 6H: lane 5′ vs. lane 5; Figure 6J). Taken together, we demonstrated that NAC promotes the autophagic degradation of eEF1A1 during CVB3 infection.

## 4. Discussion

CVB is one of the causative pathogens of myocarditis. Although most CVB-infected individuals can completely recover, viral persistence in the myocardium has been reported after the acute phase of myocarditis [2]. It has been shown that eliminating viral genomes markedly improved cardiac function [46]. Therefore, to control myocarditis progression, effective antiviral therapy is critical. Here, we investigated the effect of NAC on CVB3 replication and viral myocarditis. We demonstrated that NAC exhibits a potent anti-CVB3 effect by promoting the autophagic degradation of eEF1A1.

NAC has been used in clinical practice for a variety of conditions, such as acetaminophen overdose [47], chronic obstructive pulmonary disease (COPD) [48], and diabetes [49]. During the COVID-19 pandemic, it was proposed that NAC could inhibit COVID-19 [32]. Our previous study has shown that NAC inhibits CVB3 replication and alleviates viral myocarditis, and the antiviral mechanism of NAC is associated with its downregulation of caspase-1 [16]. Because of the well-established safety profile and the multifaceted pharmacological activities of NAC in clinical application [29], we proposed that it is worthwhile to further reveal the biological basis for the antiviral effect of NAC.

Consistent with our previous study [16], we demonstrated here that NAC shows a potent anti-CVB3 effect both in vitro and in vivo. With NAC treatment, the production of viral RNA, 3D^pol^, and viral particles was significantly decreased. Importantly, the myocardial inflammatory injury was markedly alleviated with significantly improved mouse survival and reduced virus load. We further show that the antiviral mechanism of NAC is correlated to the downregulated eEF1A1, resulting from both its decreased transcription and the increased degradation through autophagy.

eEF1A (eEF1A1 and eEF1A2) play an essential role in protein synthesis by delivering the aminoacylated tRNA to the A site of ribosome during translation elongation [40]. Increasing evidence has shown that eEF1A also exerts chaperone-like activity and participates in multiple cellular processes [37]. In this study, we show that eEF1A1 knockdown suppressed CVB3 replication. It seems unsurprising that translation inhibition, such as the depletion of eEF1A1, would show an antiviral effect since viruses usurp cellular translation machinery to synthesize viral proteins. In the present study, when eEF1A1 knockdown was performed, cell viability was carefully monitored to avoid the negative effect of the translation inhibiting of eEF1A1 siRNA. Moreover, our results show that eEF1A2, which is highly homologous to eEF1A1, was abundant in both HeLa cells and the myocardium infected with CVB3 regardless of NAC treatment. These data suggested that when eEF1A1 was knocked down, its role as an elongation factor was unlikely to be interfered with due to the presence of eEF1A2. Therefore, the global translation inhibition, if there is any, due to eEF1A1 reduction induced by NAC treatment is unlikely to contribute to the antiviral effect of NAC.

In agreement with our previous study [39], we show that CVB3 infection upregulated eEF1A1 expression in both cultured cells and the myocardium, while eEF1A2 was unchanged. Although eEF1A1 and eEF1A2 are highly homologous in the amino acid sequence and play the same role during translation elongation, their presence in normal eukaryotic cells is mutually exclusive and subjected to developmental regulation [50]. eEF1A1 and eEF1A2 are encoded in separate genes, *EEF1A1* and *EEF1A2*, which are located in different chromosomes. The expression of *EEF1A1* is almost ubiquitous, while the expression of *EEF1A2* is tissue-specific, which is limited in neurons, myotubes, and cardiomyocytes [40]. In addition, eEF1A1 is ubiquitously expressed during embryonic development [36]. In agreement with the previous studies, we show that it is eEF1A2, but not eEF1A1, that is expressed in the myocardia of either adult or newborn mice from both strains (C57BL or Balb/c), while eEF1A1 is expressed in the myocardia of Balb/c newborn mice. Moreover, CVB3 infection upregulated the expression of eEF1A1 in the mouse myocardium. Therefore, we proposed the hypothesis that eEF1A1 facilitates CVB3 infection.

We also show that eEF1A1 knockdown suppressed CVB3 replication. These data indicate that eEF1A1 was selectively upregulated to facilitate CVB3 infection, while how eEF1A1 is involved in CVB3 replication remains to be investigated. Accumulating evidence shows that, in addition to acting as elongation factors in translation, eEF1A1 also function as RNA-binding proteins (RBPs) or molecular chaperones and participate in various physiological and pathological processes of the cell, such as cell proliferation, cell death, and signal transduction [51]. It is well-established that picornaviruses rely on cellular RBPs to synthesize viral proteins and transcribe viral RNA [52]. eEF1A has been reported to be involved in the infection of a variety of DNA and RNA viruses such as DENV [53], WNV [54], and severe acute respiratory syndrome coronavirus (SARS-CoV-2) with a distinct molecular mechanism [55]. eEF1A has been identified in the purified virions of numerous viruses, such as HIV [56], SARS-CoV-2, and vesicular stomatitis virus. Further study is needed to reveal the role played by eEF1A1 in the pathogenesis of CVB or other picornaviruses.

Autophagy is a constitutive cellular process that maintains homeostasis by degrading damaged organelles and aggregated proteins [57]. It is established that picornaviruses subvert autophagy to promote viral replication [17]. CVB infection induces the formation of autophagosomes to generate viral replication membranes and blocks the fusion of autophagosomes with lysosomes [18]. Inhibiting autophagic degradation is crucial for the replication of CVB and most other viruses [18,19]. On the other hand, upregulating a complete degradative autophagy may have an inhibitory effect on viral replication. The impact exhibited by NAC on autophagy remains to be elucidated. Our results show that NAC promotes autophagy in the cells with or without CVB3 infection, suggesting that promoting autophagy might be one of the intrinsic pharmacological activities of NAC, while the mechanism involved in the upregulation of autophagy remains to be studied, likely due to its impact on mTOR or AMPK [58,59,60]. Our data suggest that complete degradative autophagy shows an antiviral effect against CVB3 infection.

We also show that eEF1A1 was degraded through autophagy by interacting with the autophagic adaptor, p62. To our knowledge, this is the first report concerning the degradation of eEF1A1 through macroautophagy. Our previous study showed that eEF1A1, which interacts with the heat shock cognate protein 70 (Hsp70), is degraded through chaperone-mediated autophagy (CMA) [39]. Our study also demonstrated that the degradation of eEF1A1 does not rely on UPS. Therefore, we conclude that the degradation of eEF1A1 is regulated by both macroautophagy and CMA. Further study is needed to show whether or not the anti-CVB effect of NAC is also involved in promoting the degradation of eEF1A1 through CMA. Proteins to be selected for autophagic degradation are often ubiquitinated [61]. p62 plays a central role as an adaptor for mediating the autophagic degradation of the substrate proteins [45]. It has been reported that the combined administration of insulin and NAC showed myocardial protection for canines with diabetes by promoting the linear ubiquitination of receptor-interacting protein kinase 1 (RIPK1) and NF-κB-essential modulator (NEMO) [62]. However, it is unclear whether or not NAC enhances the interaction between eEF1A1 and p62 by modulating the ubiquitination of eEF1A1 in the context of CVB3 infection.

In summary, we demonstrated that NAC shows a potent antiviral effect against CVB3 infection by downregulating eEF1A1.

## Data Availability

Data can be provided on request.

## Supplementary Materials

The following supporting information can be downloaded at: https://www.scidb.cn/anonymous/RW5hdXFp.
